# Clinical efficacy of supplementing qi dispelling wind and activating blood circulation method in the treatment of IgA nephropathy: A meta-analysis

**DOI:** 10.1097/MD.0000000000033123

**Published:** 2023-03-10

**Authors:** Zhiyu Pan, Mingming Zhao, Meiying Chang, Xiujie Shi, Sijia Ma, Yu Zhang

**Affiliations:** a Beijing University of Chinese Medicine, Beijing, China; b Department of Nephrology, Xiyuan Hospital of China Academy of Chinese Medical Sciences, Beijing, China; c China Academy of Chinese Medical Sciences, Beijing, China; d China Academy of Chinese Medical Sciences, Beijing, China; e China Academy of Chinese Medical Sciences, Beijing, China.

**Keywords:** activating blood circulation, Chinese medicine, dispelling wind, IgA nephropathy, meta-analysis, supplementing qi

## Abstract

IgA nephropathy (IgAN) is a common primary glomerular disease, and supplementing qi dispelling wind and activating blood is commonly used as a treatment method in Chinese medicine. However, the existing studies have small sample sizes. This study aimed to use a meta-analysis to explore the clinical efficacy of this method and to systematically introduce this effective treatment.

**Methods:**

We searched for randomized controlled trial studies on supplementing qi dispelling wind and activating blood circulation methods for IgAN indexed in the China National Knowledge Infrastructure, Wanfang Data, Chongqing VIP, SinoMed, PubMed, EMBASE, and Web of Science databases, which were interrogated from database inception to January 2022. Combining the inclusion and exclusion criteria to screen the literature, we included a total of 15 eligible studies; the quality of the included studies was evaluated using the risk of bias assessment tool of the Cochrane System Revies Manual 5.4. The outcome indexes were extracted, and a meta-analysis was performed using Review Manager 5.4 software.

**Results:**

Fifteen articles were included in this review. A meta-analysis of the results led to the conclusion that supplementing qi dispelling wind and activating blood circulation prescription has beneficial effects on the total effective rate [odds ratios = 3.95, 95% confidence interval (CI) 2.76–5.67], and can reduce 24-hour urinary protein quantity (mean deviation = −0.35, 95% CI −0.54 to −0.16) and serum creatinine (mean deviation = −15.41,95% CI −28.39 to −2.44) without impact normal level of alanine transaminase, hemoglobin, and serum albumin.

**Conclusions:**

Supplementing qi dispelling wind and activating blood can significantly improve renal function and reduce 24-hour urinary protein quantity levels in patients with IgAN compared to the use of non-Chinese medicine treatment. This finding provides a rationale for using this method in the clinical treatment of IgAN.

## 1. Introduction

IgA nephropathy is the most common primary glomerular disease in the world, accounting for 47.5% to 52.66% of all glomerular diseases and has a significant increasing trend.^[1]^Most patients follow an asymptomatic to less symptomatic course and GFR loss.^[2]^ Up to 40% of these patients eventually develop end-stage renal failure within 10 to 20 years.^[3]^ Studies have shown that the lower the level of proteinuria in patients with IgA nephropathy, the lower the risk of end-stage renal disease.^[4–6]^ As mentioned in the 2021 kidney disease: improving global outcomes guidelines, patients with 0.75 to 1g/day have a higher risk of renal function decline, and the current treatment for IgA nephropathy is mainly renin-angiotensin-aldosterone system (RAAS) blockade.^[7]^Long-term clinical practice has shown that Chinese medicine has advantages in combination with conventional treatment for IgA nephropathy. It can not only reduce proteinuria and protect renal function but also reduce the occurrence of adverse events.^[8–10]^ Supplementing qi dispelling wind and activating blood method (YQH method) is widely used in the Chinese treatment of IgA nephropathy; however, due to the small sample sizes and different research focuses,^[11,12]^ it is difficult to make accurate judgments on the therapeutic effects of YQH prescription in the treatment of IgA nephropathy. Hence, to further identify the therapeutic effects of YQH prescription on IgA nephropathy, 15 randomized controlled trials (RCTs) were selected using this method, and a meta-analysis was conducted to provide a theoretical basis for the treatment of IgA nephropathy using YQH prescription.

## 2. Methods

### 2.1. Literature retrieval

We performed a literature search of PubMed, EMBASE, Web of Sciences, China Biology Medicine disc (CBM), China National Knowledge Infrastructure, VIP, and Wanfang Data. Relevant articles were searched using the following search terms: “IgA Nephropathy” or “Berger’s Disease” and “Chinese Medicine” and “Clinical Trial.”

### 2.2. Literature inclusion and exclusion criteria

#### 2.2.1. Inclusion criteria.

(i) All participants had a clinicopathological diagnosis of IgA nephropathy.
(ii) The control group was treated with non-Chinese medicine treatment, and the treatment group was treated with YQH prescription based on control group.
(iii) RCT studies were included, regardless of whether they were double-blind.
(iv) The dosage of the YQH prescription was not limited.

#### 2.2.2. Exclusion criteria.

(i) Literature without complete information, and outcome measurements were not reported.
(ii) Animal-based research.
(iii) The specific composition of the prescriptions was unknown, and.
(iv) Research with an imbalance between the groups.

### 2.3. Literature screening

Two researchers independently screened the studies based on the inclusion criteria. The titles and abstracts were first read to exclude studies that did not meet the inclusion criteria. If a disagreement was encountered, the decision was made at the discretion of a third expert.

### 2.4. Data extraction

First author, year of publication, sample age, gender, treatment regimens for the treatment and control groups, treatment period, outcome measurements, composing of YQH prescription. All data were cross-checked and transferred to the RevMan software (5.4).

### 2.5. Quality assessment

Two reviewers independently used the Cochrane Handbook for Systematic Reviews of Interventions to evaluate the risk of bias of the included studies.

The criteria were as follows: Random sequence generation; Allocation concealment; Blinding of participants and personnel; Blinding of outcome assessment; Incomplete outcome data; Selective reporting, and; Other bias. Each criterion has 3 degrees: low, high, and unclear risk of bias.

### 2.6. Statistical analysis

Outcome indicators were analyzed using Review Manager (5.4) provided by the Cochrane Collaboration Software, and heterogeneity was assessed using the Chi-square test. Heterogeneity analysis results of *P* ≤ .05 or *I*²≥50% indicated the presence of heterogeneity in multiple independent studies, which were analyzed using a random effects model. Heterogeneity test results of *P* > .05 or *I*² < 50% indicated that there was no heterogeneity in multiple independent trials, and a fixed effect model was adopted. Funnel plots were used to assess potential reporting bias when more than 10 eligible studies were included. Count data was expressed as 95% confidence interval (CI) and odds ratios (OR). A sensitivity analysis was conducted to assess the robustness of the combined effects of the included studies.

## 3. Results

**Figure 1.**
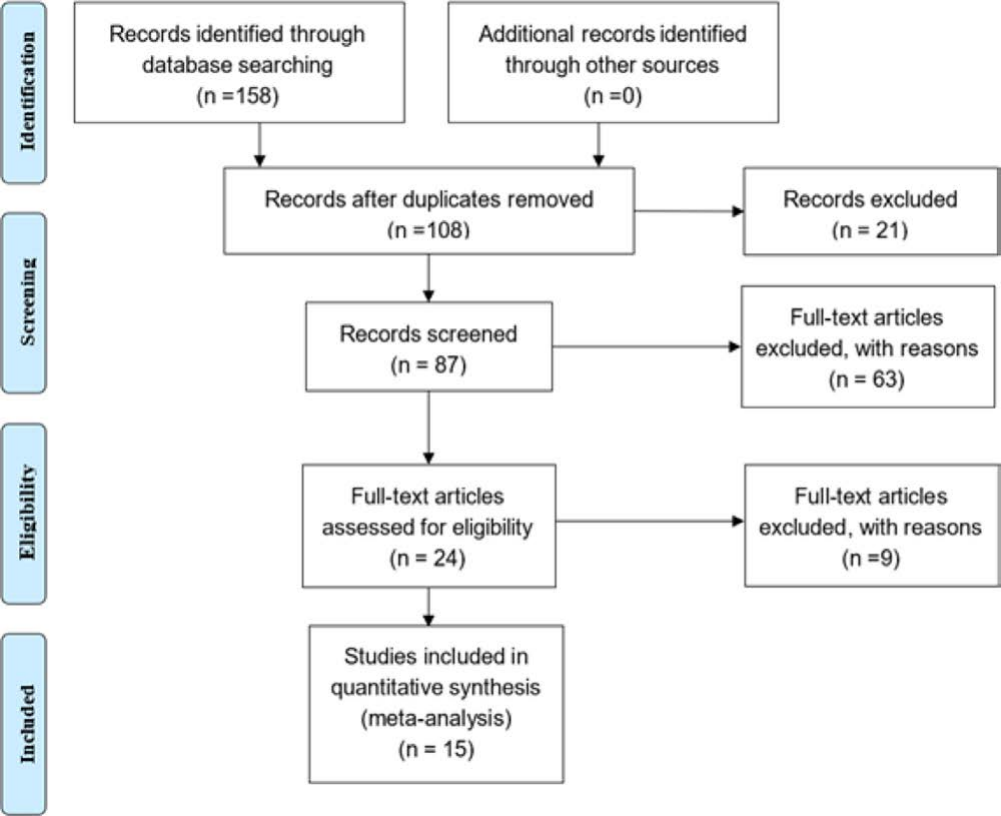
The literature screening process.

Literature search: A total of 158 studies were identified from 7 English and Chinese databases (Fig. 1). After removing the duplicates, 108 articles were selected. After careful reading of the abstracts, 87 articles were kept based on the inclusion. We retrieved and reviewed the full-text articles. Sixty-three studies were excluded owing to factors such as duplicate publications and inability to obtain available data. Twenty-four RCTs of them were eligible. Nine articles were excluded because they did not provide a complete drug composition or had an inappropriate control group design. Fifteen eligible randomized controlled studies were included in this meta-analysis. The characteristics of the selected studies are presented in Table 1, and the prescriptions are presented in the Supplemental Digital Content, see table, Supplemental Digital Content http://links.lww.com/MD/I558, which demonstrates the medicine composition of the included prescriptions.

**Table 1:** Basic information of the included literature.

First author	Years	Number of samples (E/C)	Average age (E/C)	Experimental group	Control group	Treatment duration	Outcome indexes
Han^[13]^	2012	26/26	37.04/35.33	TCM + conventional treatment	conventional treatment	3 mo	1/2/3/4
Luo^[14]^	2008	30/30	31.3/29.6	TCM + conventional treatment	conventional treatment	6 mo	1/2/4
Meng^[15]^	2011	68/56	28.3/26.6	TCM + conventional treatment	conventional treatment	6 mo	2
Pan^[16]^	2014	30/30	30.67 ± 1.51/33.9 ± 1.99	TCM + conventional treatment	conventional treatment	3 mo	2/3/5/6
Wu^[17]^	2015	30/30	35.7/35.7	TCM + conventional treatment	conventional treatment	6 mo	1/2/3/5/7
Dong^[18]^	2019	34/34	39 ± 12/38 ± 13	TCM + conventional treatment	conventional treatment	12 mo	2/3/6/7/8
Chen^[19]^	2008	32/32	30 ± 21.33/28 ± 20.24	TCM + conventional treatment	conventional treatment	6 mo	2/3/7
Chu^[20]^	2014	34/34	28.71 ± 8.99/29.2 ± 10.0	TCM + conventional treatment	conventional treatment	1mo	1/2
Xiang^[21]^	2011	28/26	29.3 ± 4.95/29.57 ± 6.17	TCM + conventional treatment	conventional treatment	8 wk	2
Yang^[22]^	2016	30/29	33.4/31.7	TCM + conventional treatment	conventional treatment	8 wk	2/3/4/5
Cai^[23]^	2020	64/63	39.41 ± 2.14/39.72 ± 2.31	TCM + conventional treatment	conventional treatment	12 mo	2/3
Chang^[24]^	2020	29/29	37.34 ± 6.77/39.03 ± 9.14	TCM + conventional treatment	conventional treatment	16 wk	2/3/6/7/8
Wang^[25]^	2021	25/27	37.84 ± 6.55/39.96 ± 9.29	TCM + conventional treatment	conventional treatment	24 wk	2/3/5/6/7/8
Yu^[26]^	2021	25/27	38.56 ± 6.25/40.74 ± 9.2	TCM + conventional treatment	conventional treatment	24 wk	5/6/7/8
Zhang^[27]^	2011	29/29	33.9 ± 8.16/31.9 ± 8.31	TCM + conventional treatment	conventional treatment	8 wk	1/2

1, Urine sediment erythrocyte count (RBC-M) (HPF); 2, 24-hour urine protein quantification (24hPRO) (g/24 h); 3, serum creatinine (SCr) (μmol/L); 4, endogenous creatinine clearance (CCr) (mL/min/1.73m^2^); 5, blood urea nitrogen (BUN) (mmol/L); 6, alanine transaminase (ALT) (U/L); 7, serum albumin (Alb) (g/L); 8, hemoglobin (Hb) (g/L).

24hPRO = 24-hour urine protein quantification, Alb = serum albumin, ALT = alanine transaminase, BUN = blood urea nitrogen, CCr = endogenous creatinine clearance, Hb = hemoglobin, RBC-M = urine sediment erythrocyte count, SCr = serum creatinine, TCM = traditional Chinese medicine.

### 3.1. Quality evaluation of the included literature

**Figure 2.**
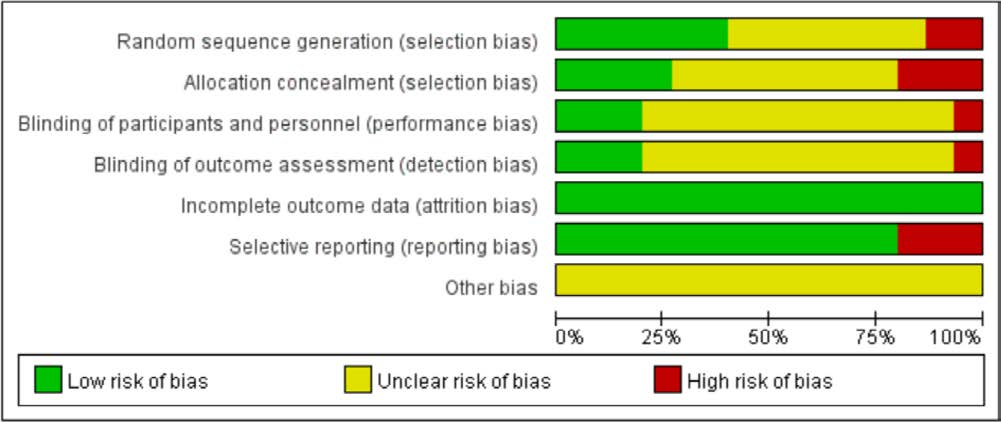
Bar chart of the risk of bias assessment of the included literature.

**Figure 3.**
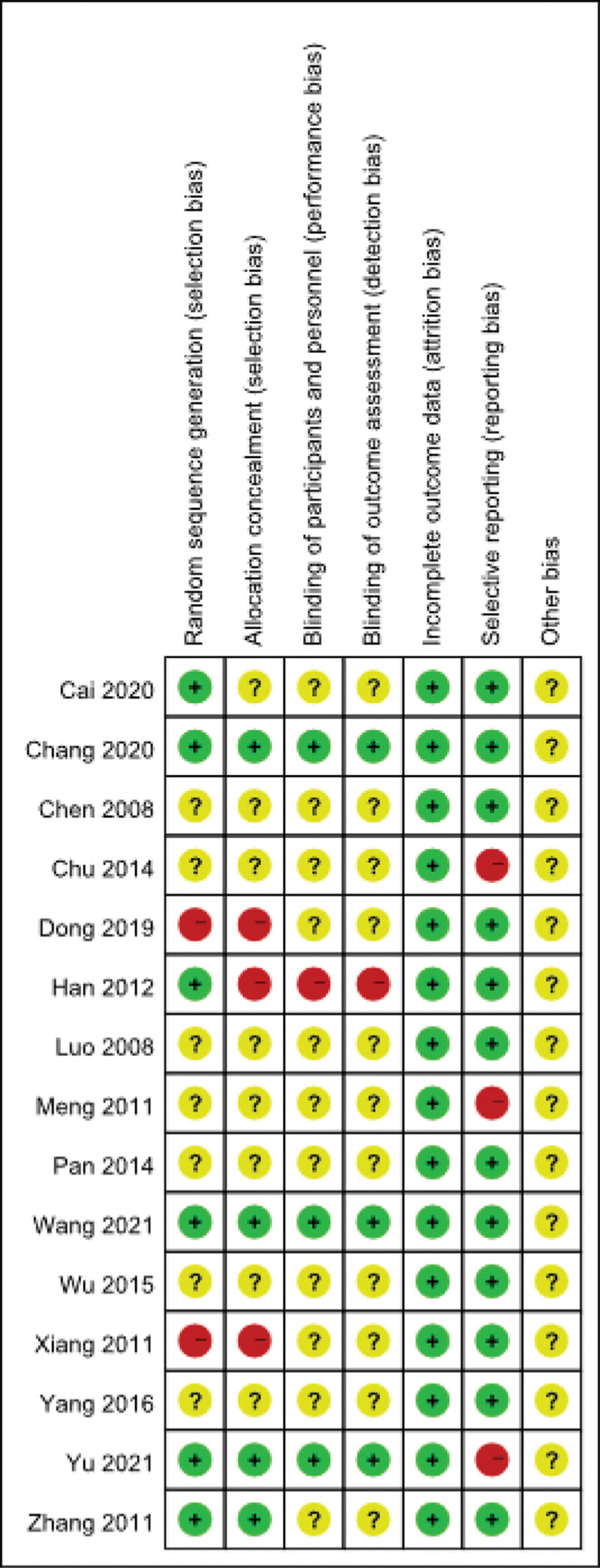
Risk of bias assessment of the included literature.

In terms of random assignment, 7 of the 15 studies were classified as unclear risk because they only mentioned “random.,”^[13–19]^ and 2 studies were considered high risk.^[20,21]^ Only 4 studies were rated as low risk due to allocation concealment.^[22–25]^ Three of the 15 studies reported a blinded process; they were rated as low risk.^[22–24]^ All 15 studies were rated as low risk due to the availability of complete outcome data. Only 3 studies reported bias, they were rated as high risk.^[14,18,24]^ The other bias was categorized as unclear because of insufficient information to assess the risk. The quality evaluation of the included articles was dominated by low risk scores. However, due to the proportion of unclear risk, and the presence of high risk items which are mainly found in allocation concealment and selective reporting. The overall quality of the studies included in this review were not high, respectively (Figs. 2 and 3).

### 3.2. Data synthesis

#### 3.2.1. Total effective rate.

**Figure 4.**
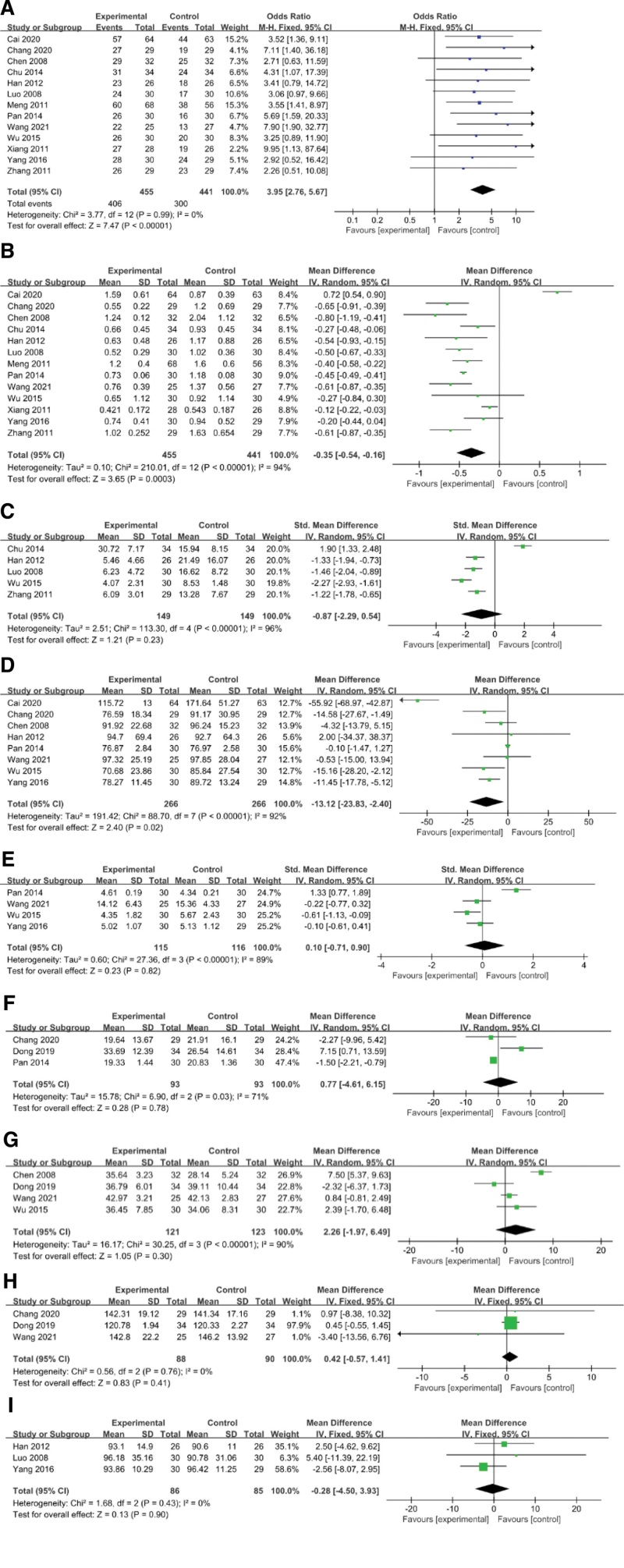
Forest plot showing the effect of YQH method for IgA nephropathy. (A) Total effective rate. (B) 24hPRO. (C) RBC-M. (D) SCr. (E) BUN. (F) ALT. (G) ALB. (H) Hb. (I) CCr. 24hPRO = 24-hour urinary protein quantity, BUN = blood urea nitrogen, CCr = endogenous creatinine clearance, Hb = hemoglobin, RBC-M = urine sediment erythrocyte count, SCr = serum creatinine, YQH method = Supplementing qi dispelling wind and activating blood method.

Thirteen studies reported the total effective rate of YQH methods in patients with IgA nephropathy.^[13–19,21–23,25–27]^ Forest plots obtained from the meta-analysis showed an OR of 3.95, 95% CI of 2.76 to 5.67, and *I*²=0%, which indicates that the overall effectiveness of the experimental group was significantly higher than that of the control group. (Fig. 4A)

#### 3.2.2. 24-hour urinary protein quantity (24hPRO).

Thirteen articles reported changes in 24hPRO^[13–19,21–23,25–27]^ with an effect size of mean deviation (MD) = −0.35, 95% CI −0.54 to −0.16 based on forest plots, and a test for effect size of Z = 3.65, *P* = .0003; heterogeneity analysis of (*P* < .00001), *I*²=94%, which suggests heterogeneity in the 13 articles and the need for a random effect model. The aggregated results of 13 RCTs suggested that YQH prescription showed favorable effects for decreasing 24hPRO in IgA nephropathy. (Fig. 4B)

#### 3.2.3. Urine sediment erythrocyte count (RBC-M).

According to the RBC-M changes in the forest plot, the effect size of the 5 selected trials^[13,16,18,25,26]^ was MD = −0.87 and 95% CI −2.29 to 0.54; the test of the effect size was Z = 1.21, *P* = .23; and the heterogeneity analysis (*P* < .00001), *I*²=96% suggests that there is heterogeneity in 5 articles; therefore, a random effect model was used. This illustrates that the RBC-M levels of the experimental group were not notably different from those of the control group (*P* > .05). (Fig. 4C)

#### 3.2.4. Serum creatinine (SCr).

Eight articles reported changes in SCr^[15–17,19,22,23,26,27]^ with an effect size of MD = −13.12, 95% CI −23.83 to −2.40 based on forest plots, and a test for effect size of Z = 2.4 and *P* = .02; heterogeneity analysis of (*P* < .00001), *I*²=92%, which suggests heterogeneity in the 8 articles and the need for a random effect model. The aggregated results of 8 RCTs suggested that YQH prescription showed favorable effects in decreasing serum creatinine of IgA nephropathy. (Fig. 4D)

#### 3.2.5. Blood urea nitrogen (BUN).

According to the BUN changes in the forest plot, the effect size of the 4 selected trials^[15,16,19,23]^ was MD = 0.10, 95% CI −0.71 to 0.90, the test of the effect size was Z = 0.23 and *P* = .82, and heterogeneity analysis (*P* < .00001), *I*²=89% suggests that there is heterogeneity in 4 articles; therefore, a random effect model was used. This illustrates that the BUN levels of the experimental group were not notably different from those of the control group (*P* > .05). (Fig. 4E)

#### 3.2.6. Alanine transaminase (ALT).

Three articles reported changes in ALT^[15,20,22]^ with an effect size of MD = 0.77 and 95% CI −4.61 to 6.15 based on forest plots, and a test for effect size of Z = 0.28 and *P* = .78; heterogeneity analysis of (*P* < .03), *I*²=71%, which suggests heterogeneity in the 3 articles and the need for a random effect model. This illustrates that the ALT levels of the experimental group were not notably different from those of the control group. (Fig. 4F)

#### 3.2.7. ALB.

According to the ALB changes in the forest plot, the effect size of the 4 selected trials^[16,17,20,23]^ was MD = 2.26 and 95% CI −1.97 to 6.49; the test of the effect size was Z = 1.05 and *P* = .30; and the heterogeneity analysis (*P* < .00001), *I*²=90%, suggests that there is heterogeneity in 4 articles; therefore, a random effect model was used. This illustrates that the ALB levels in the experimental group were not notably different from those of the control group (*P* > .05). (Fig. 4G)

#### 3.2.8. Hemoglobin (Hb).

Three articles reported changes in ALT^[20,22,23]^ with an effect size of MD = 0.42 and 95% CI −0.57 to 1.41 base on forest plots, and a test for effect size of Z = 0.83 and *P* = .41; heterogeneity analysis of (*P* = .76), *I*²=0%, which suggests no heterogeneity in the 3 articles and the need for a fixed effect model. This illustrates that the Hb levels of the experimental group were not notably different from those of the control group (Fig. 4H).

#### 3.2.9. Endogenous creatinine clearance (CCr).

According to the CCr changes in the forest plot, the effect size of the 3 selected trials^[13,19,26]^ was MD = −0.28 and 95% CI −4.50 to 3.93; the test of the effect size was Z = 0.13, *P* = .90; and the heterogeneity analysis (*P* = .43), *I*²=0% suggests that there is no heterogeneity in 3 articles; therefore, a fixed effect model was used. This illustrates that the CCr levels of the experimental group were not notably different from those of the control group (*P* > .05). (Fig. 4I)

#### 3.2.10. Publication bias.

**Figure 5.**
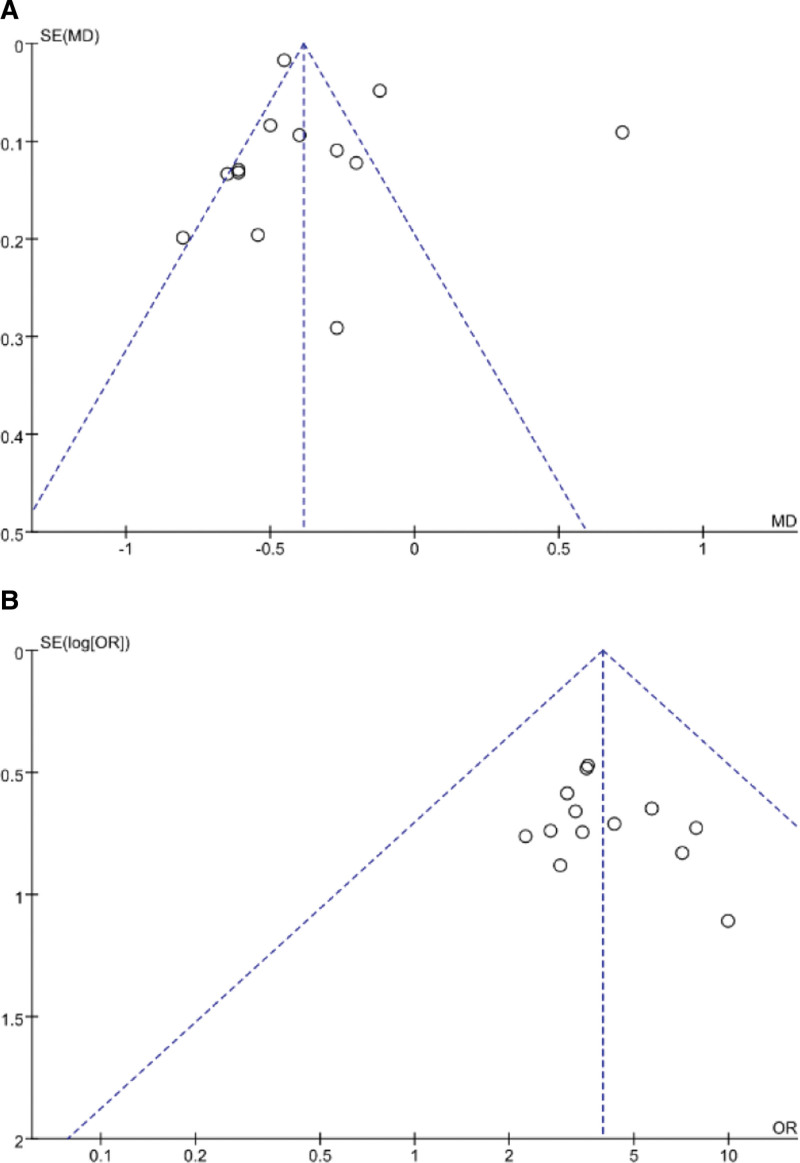
Funnel plots for publication bias. (A) 24hPRO. (B) Total effective rate. 24hPRO = 24-hour urinary protein quantity, ALT = alanine transaminase.

Funnel plots were used to measure publication bias, and 24hPRO and total effective rate had > 10 articles with an asymmetric distribution of their funnel plots, indicating possible publication bias. (Fig. 5)

## 4. Discussion

IgA nephropathy is an immune complex-mediated primary glomerular disease characterized by IgA deposition in the mesangial region and proliferation of mesangial cells. It is the most common type of chronic nephritis, with clinical manifestations of mild to moderate proteinuria and microscopic hematuria.^[28]^ Approximately 40% of patients develop end stage renal disease after the diagnosis of IgA nephropathy,^[3]^ and persistent unremitting proteinuria is a risk factor for the deterioration of renal function in patients with IgA nephropathy.^[4–6]^ kidney disease: improving global outcomes recommends the use of basic therapies, such as RAAS blockers to control blood pressure, including angiotensin converting enzyme inhibitors or angiotensin receptor antagonists, for IgA nephropathy with 24 hours urine protein quantification > 1 g.^[7]^ However, in clinical practice, angiotensin converting enzyme inhibitors and angiotensin receptor antagonists have limited efficacy in reducing proteinuria in IgA nephropathy,^[7,29]^ the results are not as good as they should be. Many clinical patients with IgA nephropathy have difficulty achieving complete remission of proteinuria after treatment with RAAS blockers. Therefore, it is important to actively seek to reduce proteinuria in IgA nephropathy based on conventional treatment in combination with Chinese medicine to stop the process of pathological damage.^[10]^

According to traditional Chinese medicine, Qi deficiency, blood stasis, and wind evil are present throughout the development of IgA nephropathy.^[30–33]^ The main pathogenesis is the Qi deficiency of spleen and kidney that is the key internal factors,^[34,35]^ and wind evil disturb the collaterals that is the initiating factor,^[33]^ and blood stasis aggravate the progression of the disease in the later stages.^[36]^ The functions of the spleen and kidney play a role in this disease, for the essence of the grain and water relies on the governing roles of them to travel through the veins and channels. If they have dysfunction, the essence of grain and water will leak out in the urine, which may manifest as proteinuria and hematuria.^[37]^ As the Inner Canon of Huangdi said, wind evil is a guide for various diseases and is characterized by opening-dispersing, migrant, and variable.^[38]^ Patients with IgA nephropathy often have cold, tonsillitis, or other illnesses at the beginning of the disease.^[39,40]^ The kidney meridians follow the throat and tongue, so wind evil can enter the kidney through the meridians and disturb the function of the kidney, resulting in foamy urine and proteinuria.^[41]^ Wind evil is often combined with other evils, such as heat and dampness. It can further damage the function of kidney, so the IgA nephropathy can be recurrent, delayed, and difficult to cure. The basic functions of the kidney are conducted by the glomerulus and tubules. The glomerulus is a mass of capillaries, and the tubules are surrounded by vessels. The collaterals of the kidney are similar to the glomerulus in structure and function.^[42,43]^ Blood stasis in collaterals can cause proteinuria and renal hypofunction, which makes the disease prolonged and difficult to treat.^[44]^

In our study, we hoped to find out the efficacy of the YQH method in the treatment of IgA nephropathy by meta-analysis. This was previously unexamined. We analyzed the results of 24hPRO, SCr, nitrogen, liver function, and albumin in the 15 included papers by RevMan, and found that the OR of total effective rate was 3.95 with 95% CI of 2.76 to 5.67; The MD value of 24hPRO was −0.35, 95% CI was −0.54 to −0.16, and the MD value of SCr was −13.12, 95% CI was −23.83 to −2.4, which means, for IgA nephropathy, the YQH method combined with conventional treatment had more significant clinical efficacy than conventional treatment used alone. However, there was no significant improvement in serum levels of BUN and CCr. The results also showed that the application of YQH method did not increase the serum level of ALT, nor does it decrease the serum level of Hb and ALB, which means that the method did not have an adverse effect on patients with IgA nephropathy. There was heterogeneity in 24-hour urine protein and serum creatinine levels, and the sources of heterogeneity may come from; Different cycles of medication; The baseline levels of the included patients were inconsistent; The drugs used in the control group were different in each study; The choice of Chinese medicine when using YQH prescription was different, and; Inconsistencies in detection methods. This study found good clinical efficacy of the YQH method in patients with IgA nephropathy, it can reduce 24hPRO and SCr without impact the normal level of ALT Hb and serum albumin. There are also limitations. For example, the sample sizes of the included studies were small, and the methodological quality of the RCTs was not high, which may have had an impact on the treatment outcome. Although the language of the study literature was not restricted, all included literature was from China, which means that the majority of all included studies were in Asian populations and multicenter large sample RCTs in different regions should be performed to complement the results of this study in the future. And the experimental design of traditional Chinese medicine treatment needs to be improved.

## 5. Conclusion

In summary, the present study showed that the combination of the YQH method was effective compared with conventional treatment used alone for IgA nephropathy. It can significantly reduce urinary protein levels, and protect kidney function without impact the serum levels of ALT Hb and serum albumin. Therefore, it can be considered as an alternative therapy to supplement traditional therapies.

## Author contributions

**Conceptualization:** Zhiyu Pan, Sijia Ma.

**Data curation:** Zhiyu Pan, Xiujie Shi.

**Formal analysis:** Zhiyu Pan.

**Investigation:** Zhiyu Pan, Mingming Zhao, Yu Zhang.

**Methodology:** Zhiyu Pan, Mingming Zhao, Yu Zhang.

**Project administration:** Meiying Chang, Yu Zhang.

**Resources:** Xiujie Shi, Yu Zhang.

**Software:** Meiying Chang.

**Supervision:** Mingming Zhao, Yu Zhang.

**Validation:** Xiujie Shi, Yu Zhang.

**Visualization:** Yu Zhang.

**Writing – original draft:** Zhiyu Pan, Meiying Chang, Sijia Ma.

**Writing – review & editing:** Zhiyu Pan.

## Supplementary Material


