# Supplementary material for: Clinical efficacy of supplementing qi dispelling wind and activating blood circulation method in the treatment of IgA nephropathy: A meta-analysis
Source: Medicine (Baltimore). 2023 Mar 10;102(10):e33123. doi: 10.1097/MD.0000000000033123 (PMC9997787; doi:10.1097/MD.0000000000033123)
Supplement: Supplementary file 1 [file medi-102-e33123-s001.pdf]

Supplemental content: Composition of experimental group prescription in the included studies.

First author	Experimental Group	Composition
Han [13]	YiQiYangYin decoction	Scutellariae Radix (Huangqin) 20g, Atractylodis Macrocephalae Rhizoma (Baizhu)15g, Saposhnikoviae Radix (Fangfeng) 15g, Ligustri Lucidi Fructus (Nüzhenzi)15g, Herba Echiptae (Hanliancao) 15g, Coicis Semen (Yiyiren)20g, Dioscoreae Rhizoma (Shanyao)15g, Chuanxiong Rhizoma (Chuanxiong)15g, Semen Nelumbinis(Lianzi)15g, Euryale ferox(Qianshi)20g, Corni Fructus (Shanzhuyu)15g, Salviae Miltiorrhizae Radix et Rhizoma (Danshen)30g, Glycyrrhizae Radix et Rhizoma (Gancao)6g.
Luo [14]	YiShenJieDu decoction	Scutellariae Radix (Huangqin)15g, Radix pseudostellariae(Taizishen)15g, Poria (Fuling)12g, Atractylodis Macrocephalae Rhizoma (Baizhu)15g, Ligustri Lucidi Fructus (Nüzhenzi)15g, Herba Echiptae (Hanliancao) 15g, Hedyotis diffusa(Baihuasheshecao)30g, Saposhnikoviae Radix (Fangfeng) 10g, cicada slough(Chantui)10g, Salviae Miltiorrhizae Radix et Rhizoma (Danshen)15g, Chuanxiong Rhizoma (Chuanxiong)10g, rhizoma imperatae(Baimaogen)30g, field thistle(Xiaoji)15g, Radix Rubiae(Qiancao)15g, Crinis Carbonisatus(Xueyutan)15g.
Meng [15]	YiShenKang pills	Scutellariae Radix (Huangqin)30g, Eucommiae Cortex (Duzhong) 15g, Hedyotis diffusa(Baihuasheshecao)25g, Euryale ferox(Qianshi)15g, Rosa laevigata(Jinyingzi)15g, Rehmanniae Radix (Shengdihuang)15g, Fructus Schisandrae Chinensis(Wuweizi)10g Herba Echiptae (Hanliancao) 15g, Saposhnikoviae Radix (Fangfeng) 15g, Atractylodis Macrocephalae Rhizoma (Baizhu)10g, Poria

		(Fuling)15g, Coicis Semen(Yiyiren)15g, Herba Leonuri(Yimucao)20g, Angelicae Sinensis Radix (Danggui)10g, Chuanxiong Rhizoma (Chuanxiong)10g, Stamen Nelmbinis(Lianxu)15g, Radix Achyranthis Bidentatae(Niuxi)10g, Glycyrrhizae Radix et Rhizoma (Gancao)3g.
Pan [16]	YiQiYangYinHuoXue decoction	Scutellariae Radix (Huangqin)30g, Rehmanniae Radix (Shengdihuang) 12g, Angelicae Sinensis Radix (Danggui)12g, Dioscoreae Rhizoma (Shanyao)15g, Atractylodis Macrocephalae Rhizoma (ChaoBaizhu)15g, Poria (Fuling)15g, Corni Fructus (Shanzhuyu)15g, Rosa laevigata(Jinyingzi)30g, Moutan Cortex (Mudanpi)12g, Herba Agrimoniae(Xianhecao)30g, Taraxaci Herba (Pugongying) 15g, Fructus Arctii(Niubangzi)15g , Saposhnikoviae Radix (Fangfeng) 10g, Salviae Miltiorrhizae Radix et Rhizoma (Danshen)10g, Glycyrrhizae Radix et Rhizoma (Gancao)6g.
Wu [17]	Not mentioned	Radix Codonopsis(Dangshen)15g, Scutellariae Radix (Huangqin)30g, Rehmanniae Radix (Shengdihuang)10g, Dioscoreae Rhizoma (Shanyao)15g, Corni Fructus (Shanzhuyu) 15g, Moutan Cortex (Mudanpi)10g, Atractylodis Macrocephalae Rhizoma (Baizhu)10g, Saposhnikoviae Radix (Fangfeng) 10g, Poria (Fuling)15g, Fructus Forsythiae(Lianqiao)15g, Radix Platycodonis(Jiegeng)10g, Radix Scrophulariae(Xuanshen)10g, Radix Ophiopogonis(Maidong)10g, Glycyrrhizae Radix et Rhizoma (Gancao)6g.
Dong [18]	YiQiQingJie decoction	Scutellariae Radix (Huangqin)30g, Atractylodis Macrocephalae Rhizoma (Baizhu)12g, Saposhnikoviae Radix (Fangfeng) 8g, Lonicerae Japonicae Flos (Jinyinhua)12g, Fructus Forsythiae(Lianqiao)12g, Angelicae Sinensis Radix (Danggui)15g, Herba Plantaginis(Cheqiancao)15g, Hedyotis

		diffusa(Baihuasheshecao)30g, Dioscoreae Nipponicae Rhizoma(Chuanshanlong)15g.
Chen [19]	GuiHuaPingFeng decoction	Scutellariae Radix (Huangqin)35g, Atractylodis Macrocephalae Rhizoma (Baizhu)10g, Saposhnikoviae Radix (Fangfeng) 10g, Hedyotis diffusa(Baihuasheshecao)25g, Angelicae Sinensis Radix (Danggui)15g, Chuanxiong Rhizoma (Chuanxiong)12g, Fructus Schisandrae Chinensis(Wuweizi)10g, Ostreae Concha (ShengMuli)15g.
Chu [20]	YiQiHuoXue decoction	Scutellariae Radix (Huangqin)30g, Atractylodis Macrocephalae Rhizoma (Baizhu)10g, Dioscoreae Rhizoma (Shanyao)15g, cicada slough(Chantui)10g, Bombyx Batryticatus(Jiangcan)10g, Curcumae Rhizoma (Ezhu) 10g, Earthworm(Dilong)10g, Radix Notoginseng(Sanqi)4g, Salviae Miltiorrhizae Radix et Rhizoma (Danshen)10g, Herba Selaginellae(Juanbai)10g, Herba seu Radix Cirsii Japonici (Daji)15g, field thistle(Xiaoji)15g, Euryale ferox(Qianshi)15g, corn silk (Yumixu)30g, Rehmanniae Radix (Shengdihuang)10g, Radix Sanguisorbae (Diyu)15g.
Xiang [21]	YiQiGuShen decoction	Scutellariae Radix (Huangqin)50g, Rehmanniae Radix (Shudihuang) 30g, Folium Perillae (Zisuye)30g, Corni Fructus (Shanzhuyu) 10g, cicada slough (Chantui)10g, Stamen Nelmbinis(Lianxu)10g, Salviae Miltiorrhizae Radix et Rhizoma (Danshen)15g, Herba Leonuri(Yimucao)15g, Rosa laevigata(Jinyingzi)30g, Euryale ferox(Qianshi)30g, Coicis Semen(Yiyiren)30g, Semen Phaseoli (Chixiaodou)30g, Glycyrrhizae Radix et Rhizoma (Gancao)5g.
Yang [22]	Not mentioned	Scutellariae Radix (Huangqin)30g, Atractylodis Macrocephalae Rhizoma (ChaoBaizhu)10g, Saposhnikoviae Radix (Fangfeng) 10g, Rhizoma Smilacis Glabrae

		(Tufuling)15g, Folium Pyrrosiae (Shiwei)15g, Rhizome Dioscoreae Hypoglaucae (Bixie)15g, Euryale ferox(Qianshi)15g, Salviae Miltiorrhizae Radix et Rhizoma (Danshen)20g, Herba Leonuri(Yimucao)20g, Angelicae Sinensis Radix (Danggui)10g, Radix Cyathulae (Chuanniuxi)15g.
Cai [23]	YiQiQingJie decoction	Taraxaci Herba (Pugongying) 30g, Scutellariae Radix (Huangqin)20g, Atractylodis Macrocephalae Rhizoma (Baizhu)20g, Lonicerae Japonicae Flos (Jinyinhua)20g, Poria (Fuling)20g, Saposhnikoviae Radix (Fangfeng) 10g, Angelicae Sinensis Radix (Danggui)10g, Paeoniae Radix Rubra (Chishao)10g, Paeoniae Radix Alba (Baishao) 10g, Chuanxiong Rhizoma (Chuanxiong)9g, Alismatis Rhizoma (Zexie)15g, Herba Plantaginis(Cheqiancao)6g.
Chang [24]	JiaWeiHuangQiChiFeng decoction	Scutellariae Radix (Huangqin)30g, Paeoniae Radix Rubra (Chishao)10g, Saposhnikoviae Radix (Fangfeng) 10g, Dioscoreae Nipponicae Rhizoma(Chuanshanlong)20g, Euryale ferox(Qianshi)20g, Rosa laevigata(Jinyingzi)10g, Hedyotis diffusa(Baihuasheshecao)20g.
Wang [25]	JiaWeiHuangQiChiFeng decoction	Scutellariae Radix (Huangqin)30g, Paeoniae Radix Rubra (Chishao)10g, Saposhnikoviae Radix (Fangfeng) 10g, Dioscoreae Nipponicae Rhizoma (Chuanshanlong)20g, Euryale ferox (Qianshi)20g, Rosa laevigata (Jinyingzi)10g, Hedyotis diffusa(Baihuasheshecao)20g.
Yu [26]	JiaWeiHuangQiChiFeng decoction	Scutellariae Radix (Huangqin)30g, Paeoniae Radix Rubra (Chishao)10g, Saposhnikoviae Radix (Fangfeng) 10g, Dioscoreae Nipponicae Rhizoma (Chuanshanlong)20g, Euryale ferox (Qianshi)20g, Rosa laevigata (Jinyingzi)10g, Hedyotis diffusa (Baihuasheshecao)20g.

Zhang [27]	YiQiZiYinHuaYuTongLuo decoction	Scutellariae Radix (ShengHuangqin)20g, Radix pseudostellariae (Taizishen)15g, Rehmanniae Radix (Shengdihuang)15g, Dioscoreae Rhizoma (Shanyao)20g, Ligustri Lucidi Fructus (Nüzhenzi)15g, Herba Echiptae (Hanliancao) 12g, Hedyotis diffusa (Baihuasheshecao)15g, Angelicae Sinensis Radix (Danggui)12g, Chuanxiong Rhizoma (Chuanxiong)15g, Paeoniae Radix Rubra (Chishao)12g, Carthami Flos (Honghua)10g, Earthworm (Dilong)12g, cicada slough (Chantui)12g, Glycyrrhizae Radix et Rhizoma (Gancao)3g.
